# Dengue virus infection-enhancement activity in neutralizing antibodies of healthy adults before dengue season as determined by using FcγR-expressing cells

**DOI:** 10.1186/s12879-017-2894-7

**Published:** 2018-01-10

**Authors:** Minh Huong Phu Ly, Meng Ling Moi, Thi Bich Hau Vu, Mya Myat Ngwe Tun, Todd Saunders, Cam Nhat Nguyen, Anh Kieu Thi Nguyen, Hung Manh Nguyen, Than Huu Dao, Do Quyen Pham, Thi Thu Thuy Nguyen, Thi Quynh Mai Le, Futoshi Hasebe, Kouichi Morita

**Affiliations:** 1 Department of Virology, Institute of Tropical Medicine, Nagasaki University, Sakamoto 1-12-4, Nagasaki, 852-8523 Japan; 2 Graduate School of Biomedical Sciences, Nagasaki University, Nagasaki, Japan; 3 Program for Nurturing Global Leaders in Tropical and Emerging Communicable Diseases, Nagasaki University, Nagasaki, Japan; 4 Department of Virology, National Institute of Hygiene and Epidemiology, Hanoi, Viet Nam; 5 Ha Noi Preventive Medicine Center, Hanoi, Viet Nam; 6 Vietnam Research Station, Center for Infectious Disease Research in Asia and Africa, Institute of Tropical Medicine, Nagasaki University, Nagasaki, Japan

**Keywords:** Dengue virus (DENV), Dengue virus neutralizing antibody, Primary DENV infection, Secondary DENV infection, Monotypic, heterotypic immune response

## Abstract

**Background:**

Antibodies are critical responses to protect the host from dengue virus(DENV) infection. Antibodies target DENV by two pathologic mechanisms: virus neutralization and infection enhancement. In dengue patients, the absence of neutralizing activity in the presence of FcγR implies that infection-enhancing activity hampers the neutralizing activity of antibodies, which could potentially lead to symptomatic presentations and severe clinical outcomes.

**Methods:**

A total of 100 pair serum samples from adult healthy volunteers were obtained during the dengue season in Ha Noi in 2015 for evaluation of neutralizing and infection-enhancing activity. Additionally, 20 serum samples from acute secondary DENV infection patients were also used as the patient group in this study. PRNT was performed on BHK cells and FcγR-expressing BHK cell lines for all serum samples.

**Results:**

Out of 100 residents, positive neutralizing antibodies (N.A) were found in 44.23 and 76.92% for DENV-1; 38.46 and 75% for DENV-2; 19.23 and 15.38% for DENV-3; and 1.92 and 9.62% for DENV-4 for pre and post-dengue season respectively. The percentage of post-exposure residents having positive responses against single, two, or more than three DENV serotypes were 38.46, 44.23 and 15.38%, respectively. A total of 34 residents were DENV seropositive before the dengue season and these individuals demonstrated further elevation of IgG antibodies after the dengue season. At the end of the season, 18 residents were confirmed to be new asymptomatic DENV infection cases. In both groups, N.A titers determined on BHK cells were higher than that on FcγR-expressing BHK cells. In heterotypic N.A responses, N.A titers to the infecting serotype from the samples obtained from pre-exposure group were significantly higher than those of the patient group. However, fold enhancement to the infecting serotypes from the samples in the pre-exposure group was substantially lower as compared to that of the patient group.

**Conclusion:**

Before and after the dengue season, serum samples from healthy volunteers demonstrated high levels of neutralizing antibodies and low or absence of infection-enhancement activity. The results suggest that while infection-enhancement activity hampers neutralizing activity of antibodies, high levels of DENV neutralizing antibodies set a critical threshold in facilitating the prevention of disease progression.

**Electronic supplementary material:**

The online version of this article (10.1186/s12879-017-2894-7) contains supplementary material, which is available to authorized users.

## Background

Dengue, currently found in 128 countries, is an important mosquito-borne viral disease posing a threat to health of many global communities [1]. The disease results from infection with dengue virus (DENV), which consists of four antigenically distinct serotypes known as DENV-1, DENV-2, DENV-3 and DENV-4 from the genus *Flavivirus* in the family *Flaviviridae.* Based on epidemiologic studies, it is believed that more than 390 million DENV infections occur every year of which approximately 96 million cases are symptomatic [2]. The burden of dengue continues unabated as the DENV serotypes expand into new areas. In the regions where dengue is endemic, whole populations are at risk. Individuals infected by any of the DENV serotypes will develop protective monotypic immunity evidenced by the generation of dengue immunoglobulin M (IgM) and immunoglobulin G (IgG) antibodies. The IgM antibodies may be found in serum as early as 4 days after the onset of disease [3]. The IgG antibodies present in serum at the end of the convalescent period (9-10 days) in primary infection and may also be detected earlier in the case of secondary infection. IgG levels are elevated up to 30-40 days after infection and neutralizing antibodies to the infecting virus last a life-time [4, 5].

Antibodies are critical responses protecting the host from DENV infection. Antibodies target DENV by two pathologic mechanisms: virus neutralization and infection enhancement. At high avidity, antibodies neutralize DENV, whereas lower level avidity of antibodies enhance DENV infection and hamper virus neutralization [6]. Primary infection with one serotype produces long-term protective immunity to re-infection with the homologous serotype. After a limited period of cross-protection, individuals having a primary DENV infection are susceptible to secondary infection with heterologous serotypes [7, 8]. Individuals exposed to secondary infections are more likely to develop severe symptoms compared to those exposed to primary infections only [9]. During secondary infection, non-neutralizing antibodies from the first infection bind to the second serotype to form DENV-antibody complexes. These immune complexes are more readily taken up by FcγR-bearing myeloid cells such as monocytes and macrophages than uncoated virus particles [8]. This effect represents antibody-dependent enhancement (ADE) phenomenon which results in higher levels of progeny virus production and has been hypothesized to lead to severe dengue [10].

A previous study using plaque reduction neutralization test (PRNT) demonstrated that neutralizing antibody titers on BHK cells were higher than those on FcγR-expressing BHK cells [6]. In patients with symptomatic dengue, the absence of neutralizing antibodies in the presence of FcγR implies that infection-enhancing activities hampers the neutralizing activity of an antibody [6]. Neutralizing antibodies are thought to provide long-term protection against DENV infection of the same serotype, while recent studies have also demonstrated an association of higher levels of neutralizing antibodies with a reduced risk of secondary DENV infection [11–13]. Additionally, high levels of neutralizing antibodies correlate with lower probability of getting symptomatic infection [14]. By using FcγR-expressing cells, we demonstrated that in dengue patients, infection-enhancement activity existed when the cross-reactive neutralizing activities was reduced [6]. However, the levels of antibodies in the presence and absence of the FcγR cell line from asymptomatic DENV infection cases are still limited. Thus, we sought to test the hypothesis that the pre-existence of neutralizing antibody titers before the dengue season are linked with protection from symptomatic infection by using serum samples from residents in a DENV hyper-endemic region before and after the dengue season. In this study, we examined the sum of neutralizing activity in the presence of infection-enhancement activity by using FcγR-expressing BHK cells.

## Methods

### Sample collection

In Viet Nam, the first case of dengue hemorrhagic fever (DHF) was identified in 1963 in the Mekong Delta region of southern Viet Nam [15]. Between 1963 and 1995, approximately 1,518,808 DHF cases with 14,133 deaths had been reported in this country [16, 17]. Major epidemics of dengue had occurred in 1969, 1983, 1987, 1998 and 2009 in which the 1987 dengue outbreak with 354,517 cases and 1566 deaths represented the single largest reported dengue outbreak in the world [18]. In 2015, 54 out of 63 provinces reported a combined 97,476 case of dengue including 61 deaths [19]. While all four DENV serotypes co-circulate in Viet Nam, climate differences in Viet Nam result in differences in dengue epidemiology across the country. In Ha Noi, the capital city located in northern Viet Nam, dengue transmission intensity is quite low, usually peaking between October and November during the hot, wet season, with the majority cases being reported in adults.

In this study, 100 paired blood samples were collected from healthy volunteer adults who resided in Ha Noi, Viet Nam. The first sampling was performed before the dengue season in August 2015. This is the pre-exposure group or pre-dengue season group. The second sampling was done at the end of the dengue season in November 2015. This is the post-exposure group or post-dengue season group. During our sampling period, the four DENV serotypes were co-circulating in the area however, DENV-1 and DENV-2 were the dominant serotypes. Sera were separated as quickly as possible, transferred to sterile 2 ml cryo-tubes and stored in −70°C until used. In this study, (1) none of the participants reported fever or symptoms/signs suggesting dengue fever at the time of recruitment, (2) none had a fever within three months of recruitment nor (3) during the approximately three-month study period.

An additional 20 serum samples obtained from acute secondary adult DENV infection patients were also used in this study (the patient group) for further analysis and comparison. The serum samples were obtained from patients within 3 days of the onset of the disease. The patient samples were negative for anti-DENV IgM antibodies but positive for anti-DENV IgG antibodies. Only samples confirmed by virus isolation and real-time polymerase chain reaction (PCR) was used for the study. Thus, the day of the onset of fever was narrowed to less than 4 days to satisfy this criteria. Due to the absence of anti-DENV IgG antibodies in the acute phase, the acute primary DENV infection cases were not used as the patient group for the purpose of comparing the neutralizing activities between patient group and non-patient group (healthy volunteers).

### Virus and cell lines

DENV-1 (99St12A strain), DENV-2 (00St22A strain), DENV-3 (SLMC50 strain), and DENV-4 (SLMC318 strain) were used for DENV IgM capture ELISA. These viruses were propagated onto C6/36 mosquito cell lines. Infected culture fluid (ICF) was collected on day 7 post-infection for the preparation of a tetravalent DENV antigen (25 ELISA units/serotype).

DENV-1, 01-44-1HuNIID strain (GenBank accession no. AB111007), DENV-2, 00St22A strain, DENV-3 5528 strain (GenBank accession no. KP893718) and DENV-4 SLMC318 strain were used for PRNT in this study as these strains form clear plaques. In PRNT experiments, virus stock was prepared by using baby hamster kidney cell lines (BHK- Japan Health Science Research Resource Bank, Japan) [6]. Infected culture fluid (ICF) collected on day 5 post-infection was used to inoculate BHK and FcγR-expressing BHK cell lines for virus titration and neutralization tests. BHK cells were cultured in Eagle’s Minimum Essential Medium (EMEM) (Sigma, USA), supplemented with heat-inactivated 10% fetal bovine serum (FBS, Sigma) at 37°C in 5% CO_2_. FcγR-expressing BHK cells were cultured in EMEM (Sigma), supplemented with heat inactivated 10% fetal bovine serum (FBS, Sigma) and 0.5 mg/ml Neomycin (G418, PAA Laboratories GmbH, Austria) at 37°C in 5% CO_2_.

### Detection of DENV IgM

Following Bundo and Igarashi’s (1985) protocol [20], an in-house DENV IgM capture ELISA (in-house IgM ELISA) was performed to confirm the presence of anti-DENV IgM antibodies in serum samples. First, the polystyrene 96-well microplates (Maxisorp Nalge Nunc International, Roskilde, Denmark) were coated with 100μl (5.5μg/100μl) of 100-time diluted goat anti-human IgG antibodies specific for IgM (μ-chain specific) (Cappel ICN Pharmaceuticals, Aurora, OH) with ELISA coating buffer (0.05 M carbonate-bicarbonate buffer, pH 9.6 containing 0.02% sodium azide) in all wells, except for the blanks at 4°C overnight. Then, to avoid non-specific binding, the wells, except for the blanks were blocked with 100 μl of undiluted Blockace Solution (UK-B 80, Yukijirushi, Sapporo, Japan), and incubated for 1 h at room temperature (RT). The microplates were washed three times with PBS containing 0.05% Tween 20 (PBS-T) (Gibco, NY, USA). Positive and negative controls and patient sera were diluted 1:100 in PBS-T and 100 μl of diluted specimen was added into duplicate wells. The plates were incubated at 37°C for 1 h and nonspecific reactants were removed by washing three times with PBS-T. A tetravalent DENV antigen (25 ELISA unit/serotype) was used as an assay antigen in this experiment. A total of 100 μl of DENV antigen was then added to each well and incubated at 37°C for 1 h. Unbound DENV antigen was then removed by washing the wells three times with PBS-T. Next, a total of 100 μl of horseradish peroxidase (HRPO)-conjugated anti-flavivirus (anti-DENV, anti JEV) mouse monoclonal antibody (12D11/7E8) [21–23] at a dilution of 1:2500 (diluent was PBS-T mixed with 10% Blockace) was added to the wells and incubated at 37°C for 1 h. If DENV antigen were retained in the wells by the anti-DENV antibodies in the serum samples, the HRPO conjugated anti-flavivirus mouse monoclonal antibody would bind to the DENV antigen in the wells. Excess conjugate was removed by washing three times with PBS-T. A total of 100 μl of substrate solution, 5 mg *o*-phenylenediamin dihydrochloride (OPD) (Sigma Chemical, St. Louis, MO) and 0.03% hydrogen peroxide in 10 ml of 0.05 M citrate-phosphate buffer, pH 5.0 was added to each well (substrate buffer was also added in blank wells) and incubated in the dark at RT for 1 h. The color was allowed to develop during this incubation time. To terminate the reaction, 100 μl of stop solution (1 N sulfuric acid) was added to each well (including the blanks) and the optical density at 492 nm (OD_492_) was measured using a Multiscan ELISA plate reader (Thermolabsystem, Tokyo, Japan). The positive control or the samples with OD_492_ two times or higher than the negative control were considered positive (OD_492_ of positive control or samples / OD_492_ of negative control ≥ 2).

### Detection of anti-DENV IgG antibodies

In the IgG assay, an in-house flavivirus IgG indirect ELISA protocol, modified by Inoue et al. (2010) [24] was used to measure DENV IgG titer. In the modified protocol, instead of DENV antigen, a total of 100 μl of purified Japanese encephalitis virus (JEV) antigen (JEV ML-17 strain; 250 ng per well in ELISA coating buffer) was used to coat the 96-well microplates (Maxisorp Nunc, Denmark) with the exception of the blanks at 4°C overnight. To avoid non-specific binding, the wells (except for the blanks) were blocked with 100 μl of undiluted Blockace and incubated for 1 h at RT. The microplates were then washed three times with PBS-T. To each well, 100 μl of serum samples at a 1:1000 dilution in PBS-T + 10% Blockace was added and incubated for 1 h at 37°C. Standard serum containing antibodies against the test antigen and the serum from the non-disease population were added on each plate for positive and negative controls, respectively. The microplates were wash three times with PBS-T. Next, 100 μl of goat anti-human IgG antibodies conjugated to horseradish peroxidase (HRPO) (American Qualex, San Clemente, CA), at a 1:25,000 dilution in PBS-T + 10% Blockace were added to each well and the plates were incubated for 1 h at 37°C. The microplates were again washed three times with PBS-T and 100 μl of OPD substrate solution was added to each well (substrate buffer was also added in the blank wells). The microplates were incubated in the dark at RT for 30 mins and 100 μl of stop solution was finally added to each well (including the blanks) to terminate the reaction. A standard curve was generated by using the OD_492_ values of 2-fold diluted dengue positive control serum from a 1:1000 dilution to 1:2^14^, in PBS-T + 10% Blockace. The IgG titers of the patient sera were determined by the positive standard curve. A sample titer equal to, or greater than, 1:3000 (cut-off value of positive IgG was at 1:1000 + 3 standard deviations) was considered IgG-positive for dengue virus [24]. To confirm that the IgG antibody detected was not confounded by the cross-reactivity to JEV, all the results obtained by the in-house flavivirus IgG indirect ELISA described above were investigated again by a commercial anti-DENV IgG kit (Dengue IgG indirect Elisa, Pan Bio, Inverness, Australia) according to the manufacturer’s instructions.

### Infection assay

Serum samples that were seropositive with both in-house flavivirus IgG indirect ELISA and Dengue IgG indirect Elisa (Pan Bio, Inverness, Australia) were checked for the presence of neutralizing antibodies to specific DENV serotypes using plaque reduction neutralization test (PRNT). The samples were serially diluted 2-fold from 1:10 to 1:2560 in EMEM/2% FBS. The serum samples were heat-inactivated at 56°C for 30 min before use. As the amount of serum sample was limited, two replicates were tested for each of the serum samples to four DENV serotypes. A total of 25 μl of virus mixture containing 35-50 plaque-forming units (35-50 PFU/25μl or 1400-2000 PFU/ml) and 25 μl of the diluted serum samples were mixed to allow virus-antibody neutralization reaction. After 1 h incubation at 37°C, 50 μl of the mixture were inoculated onto BHK cell (FcγR-negative BHK) and FcγR-expressing BHK cell monolayers in 24-well plates and incubated for 60 min at 37°C in 5% CO_2_ then overlaid with 1 ml of EMEM (Nissui Pharmaceutical, Japan) containing 2% FBS and 1% methylcellulose (Wako Pure Chemical Industries, Japan). The plates were incubated at 37°C in 5% CO_2_ for 5-7 days until plaque formation was confirmed by the naked eye. The cells were then fixed with 4% paraformaldehyde phosphate buffer solution (Wako, Osaka, Japan) for 30 min at RT and then stained with 1.25% crystal violet (Wako, Osaka, Japan). The number of plaque was counted again by naked eye. PRNT_50_ end points were calculated using the reciprocal of the final serum dilution showing a 50% or greater reduction in plaque counts in wells compared to the number of plaque from the negative control wells with no antibodies. Positive PRNT samples were defined as having a neutralizing titer of 10 or above to any of the viruses tested (PRNT_50_ ≥ 10 or Log_2_ (PRNT_50_) ≥3.32) [25]. PRNT assay using JEV was also performed in order to confirm the seropositive samples were truly DENV infection. Neutralization antibodies (N.A) against JEV from the sero-positive samples were much lower compared to N.A against DENV.

In the infection-enhancement assay, serum samples were diluted with EMEM/2% FBS. A total of 25 μl of DENV containing 35-50 plaque-forming units (35-50 PFU/25μl or 1400-2000 PFU/ml) were mixed with 25 μl of 1:20 diluted serum samples (a 1:20 dilution was used instead of a 1:10 dilution due to insufficient amount serum samples from the second sampling). The presence of ADE activity was determined by using plaque assay on FcγR-expressing BHK, which was similar to the method for the neutralizing assay. Positive infection-enhancement (measured as fold-enhancement) was defined as fold-enhancement values greater than the cut-off value plus 2 times standard deviation (cut-off value = mean plaque count at a 1:20 serum dilution on FcγR-expressing BHK)/(mean plaque count in the absence of human samples on FcγR-expressing BHK cells).

### Data analysis

In this study, all data from PRNT_50_ result was transformed to base-2 logarithm for analysis. In descriptive analyses, numbers and percentages were used for categorical variables. Mean and standard deviation (SD); median and interquartile range (IQR) were used for continuous variables. Odds ratio (OR) and the 95% confidence interval (95% C.I) were estimated to indicate the probability of DENV infection for each group. For comparison in specific groups, chi-squared test, Fisher’s exact test, Wilcoxon rank-sum (Mann-Whitney) test and t-test was used appropriately. Statistical tests were performed with Stata 14.1 (StataCorp LP, College Station, Texas 77,845 USA) and GraphPad Prism version 7.0a (GraphPad software, La Jolla California USA) with 5% level of significance and two-tailed *p* values.

## Results

### Demographic data

Among the residents, 74% (74/100) were found in the age group of 20-30 years, 22% (22/100) were 30-40 years and 4% (4/100) were more than 40 years old respectively (Table 1). Of these, 27 (27%) were male and 73 (73%) were female (Table 1). The mean age of the subjects was 28.06 years with the youngest and the oldest age being 21 and 52 respectively (Table 2).

**Table 1:** Characteristic of the study population

Variable		Total (n)	DENV IgG positive ^a^ N (%)	Crude OR	95% C.I	*p*-value
Before dengue season		100	34 ^c^ (34.00)			
Age group (years)	20–30	74	20 (58.82)	1.00		0.05
	30–40	22	12 (35.29)	3.24	1.21–8.66	
	≥40	4	2 (5.88)	2.70	0.35–20.48	
Gender	Male	27	11 (32.35)	1.00		
	Female	73	23 (67.65)	0.67	0.27–1.68	0.39
DENV IgM ^b^	Negative	94	34 ^c^ (100.00)			
	Positive	6	0 (00.00)			
After dengue season		100	52 ^d^ (52.00)			
Age group (years)	20–30	74	35 (67.31)	1.00		0.25
	30–40	22	14 (26.92)	1.95	0.73–5.20	
	≥40	4	3 (5.77)	3.34	0.33–33.63	
Gender	Male	27	19 (36.54)	1.00		0.02
	Female	73	33 (63.46)	0.34	0.13–0.92	
DENV IgM ^b^	Negative	82	40 (76.92)	1.00		
	Positive	18	12 (23.08)	2.10	0.71–6.23	0.17

*Crude OR* Crude odds ratio, *95% C.I* 95% confidence interval

^a^ DENV IgG result was as determined by using in-house DENV IgG Elisa and DENV IgG indirect Elisa (Panbio)

^b^ DENV IgM result was determined by using an in-house DENV IgM Elisa test

^c^ These 34 individuals were considered as asymptomatic secondary infection (S.I) cases

^d^ Out of 52 individuals, 34 had DENV IgG before the dengue season and continue to have elevated DENV IgG after the dengue season, the remain 18 had only DENV IgG after the dengue season

**Table 2:** Comparison of DENV IgG titer in 100 individuals before and after the dengue season

Variable		Mean	t-value	S.D	95% C.I	*P*-value
Age		28.06 (21–52)		5.41		
IgG titer pre	M (27)	6149.93		10190.48	2118.71–10181.15	
	F (73)	4679.02		9138.45	2546.86–6811.18	
	t-test		0.69			0.49
IgG titer post	M (27)	13455.84		23757.02	4057.88–22853.80	
	F (73)	7187.98		13360.80	4070.68–10305.29	
	t-test		1.66			0.10
IgG titer pre		5076.17		9404.20	3210.17–6942.17	
IgG titer post		8880.30		16907.77	5525.44–12235.17	
	t-test		2.53			0.01

IgG titer pre: IgG titer from 100 healthy individuals before exposure to the dengue season

IgG titer post: IgG titer from 100 healthy individuals after exposure to the dengue season

*S.D* Standard deviation

### Anti-DENV IgM and IgG antibody levels of the study population before and after the dengue season

All participants were screened for the presence of DENV IgM, and IgG antibody levels before and after the dengue season to determine possible exposure to DENV. Prior to the dengue season, out of 100 serum samples, 34 (34%) individuals had DENV IgG indicating past DENV infection. In the group of 34 individuals, 11 (32.35%) were male and 23 (67.65%) were female. The number of samples showing positive for DENV IgG was highest (20/34, 58.82%) in the age group 20-30 years old (Table 1). At the end of the season, of 100 serum samples collected from the same population, 52 (52%) samples showed positive for DENV IgG antibodies, in which 19 (36.54%), 33 (63.64%) were male and female respectively. Again, residents in the 20-30 year-old range were the most affected population as the percentage of individuals having DENV IgG was highest compared to other age groups (Table 1). Out of 52 individuals who were positive for DENV IgG antibodies, DENV antibody levels increased in 34 participants (who were originally IgG seropositive) after the dengue season. The remaining 18 individuals only seroconverted after the dengue season and were considered as new asymptomatic DENV infection cases (Table 1). In addition, DENV IgG titers of the post-exposure residents was significantly higher than that of pre-exposure residents (*p* = 0.01) (Table 2). Among the 34 individuals with past DENV infection, DENV IgG titers after the dengue season was also substantially higher than that of before the season (*p* = 0.015, data not shown).

An in-house DENV IgM capture ELISA test revealed that 6% (6 out of 100) of the samples were positive before the dengue season (Table 1). At the end of the dengue season, 18% (18 out of 100) of samples were DENV IgM positive. Of these, 6 samples were positive for DENV IgM only and 12 samples were positive for both DENV IgM and IgG antibodies (Table 1).

### Levels of neutralizing and infection-enhancing antibodies before and after the dengue season

**Fig. 1:**
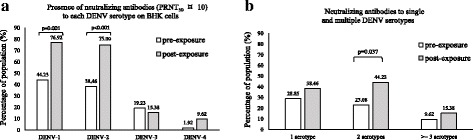
Overall analysis of neutralizing antibody titer in serum samples obtained from 100 healthy volunteer residents before (pre) and after (post) exposure to the dengue season in 2015 in Ha Noi. *P*-value determined by Fisher’s exact test. *P* value ≤0.05 = significant. Neutralizing antibody titers were determined on BHK cell line

Before the dengue season occurred, neutralizing antibodies (PRNT_50_ ≥ 10) were detected in 44.23% for DENV-1, 38.46% for DENV-2, 19.23% for DENV-3 and 1.92% for DENV-4 of the population respectively. After the dengue season, although the proportion of individuals having neutralizing antibodies for DENV-3 slightly decreased (15.38%), percentages of the population that demonstrated neutralizing antibodies to each of the DENV serotypes were higher at 76.92, 75 and 9.62% for DENV-1, DENV-2, and DENV-4, respectively (Fig. 1a). Furthermore, most of the serum samples showed higher proportions of not only monotypic antibody responses, but also heterotypic antibody responses after the dengue season. Percentage of the population having positive responses against single, two and more than three DENV serotypes after the dengue season were 38.46, 44.23 and 15.38% respectively (Fig. 1b).

Prior to the dengue season, out of 100 serum samples, 34 individuals had dengue IgG indicating past DENV infection. At the end of the dengue season, 52 of 100 samples were determined to have dengue IgG, in which 34 participants who were original seropositive before exposure to the season demonstrated further increase in the level of dengue IgG titers after the dengue season. The remaining 18 seroconverted individuals after the dengue season were considered as new asymptomatic DENV infection cases (Table 1). Two further analyses of DENV neutralizing antibody profiles will compare: (1) the 34 individuals who had past DENV infections before exposure to the dengue season in 2015 (pre-dengue season group) to the patient group, and (2) the 18 new asymptomatic DENV infection cases (post-dengue season group) to the same patient group.

### Neutralizing and infection-enhancing activities of the 34 individuals with elevated anti-DENV IgG levels after dengue season

DENV neutralizing antibody titers (N.A titers) of 68 serum samples obtained from 34 individuals (34 paired serum samples) with elevated levels of dengue IgG were examined using BHK (FcγR negative BHK) and FcγR-expressing BHK cell lines. For the purpose of analysis, the means of the N.A titers to specific DENV serotype described below were the absolute mean of Log_2_ (N.A titers). The means of N.A titers on BHK cells and FcγR-expressing BHK cells from serum samples in the pre-dengue season group (labeled as Non patient 1 in Additional file 1: Figures S1A, S1B) were 4.59 ± 0.37 and 3.41 ± 0.29 for DENV-1; 4.29 ± 0.33 and 3.2 ± 0.23 for DENV-2; 3.06 ± 0.23 and 2.5 ± 0.11 for DENV-3; 2.35 ± 0.03 and 2.32 ± 0 for DENV-4. The means of N.A titers as determined by BHK and FcγR-expressing BHK cells from serum samples after the dengue season (labeled as Non patient 2 in Additional file 1: Figures S1A, S1B) were 4.76 ± 0.34 and 3.65 ± 0.3 for DENV-1; 4.79 ± 0.32 and 3.26 ± 0.24 for DENV-2; 2.97 ± 0.28 and 2.65 ± 0.17 for DENV-3; 2.53 ± 0.1 and 2.32 ± 0 for DENV-4. In contrast, the means of N.A titers as determined by BHK cells and FcγR-expressing BHK cells using serum samples from dengue patients (labeled as Patient in Additional file 1: Figures S1A, S1B) were 4.47 ± 0.36 and 3.17 ± 0.35 for DENV-1; 4.67 ± 0.2 and 3.12 ± 0.22 for DENV-2; 3.22 ± 0.31 and 2.67 ± 0.21 for DENV-3; 2.62 ± 0.15 and 2.32 ± 0 for DENV-4. Overall, regardless the origin of the serum samples (either from Non-patient groups or Patient group) N.A. titers to specific DENV serotype determined by BHK cells were higher than those determined by FcγR-expressing BHK cells (Additional file 1: Figure S1), and in some serum samples, neutralizing antibodies were not detected when FcγR-expressing BHK cells were used as assay cells (Additional file 2: Table S1).

**Fig. 2:**
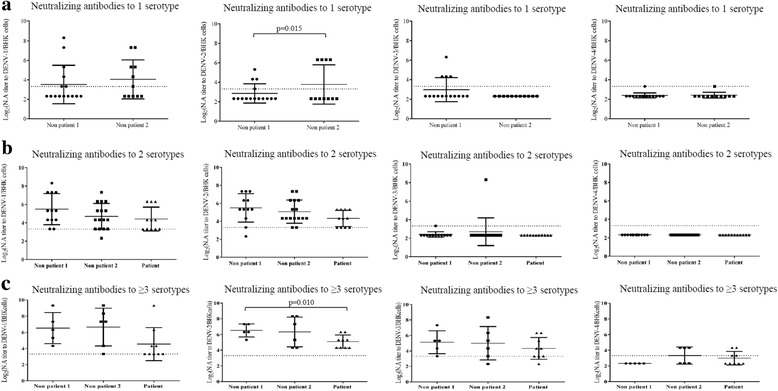
Neutralizing antibody titer reacts to single or multiple DENV serotypes in 34 pair serum samples obtained from 34 individuals who were DENV IgG positive before the dengue season and demonstrated elevated DENV IgG after the season versus patient group. Neutralizing activity was determined by PRNT_50_ on BHK cells. Positive PRNT samples were defined as having a neutralizing titer ≥10 or Log_2_(PRNT_50_) ≥3.32 to any of the virus test (above dotted line). Non patient 1 = pre-dengue season group; Non patient 2 = post-dengue season group; Patient = confirmed DENV infected patient group. *P*-value was determined by t-test. *P* value ≤0.05 = significant. Comparison was performed between pre-dengue season group (Non patient 1) vs. patient group. The results were represented as means of Log_2_(N.A titers) ± standard deviation (Mean ± SD)

We then compared the levels of neutralizing antibody (N.A) titers to each serotype in individuals with homotypic and heterotypic antibody responses. In homotypic antibody responses (N.A reacted to one/single DENV serotype, Fig. 2a), the means of N.A titers for pre-dengue season group (Non patient 1) and post-dengue season group (Non patient 2) were 3.52 ± 0.51 and 4.05 ± 0.6 for DENV-1, 2.86 ± 0.26 and 3.78 ± 0.61 for DENV-2 (*p* = 0.015), 2.98 ± 0.32 and 2.32 ± 0 for DENV-3, 2.32 ± 0.31 and 2.32 ± 0.31 for DENV-4. In the patient group, although serum samples were collected at the acute phase, none of them demonstrated reactivity against a single serotype (Fig. 2a). Heterotypic antibody responses were found in all subsets (Fig. 2b, c). The means of N.A titers were high in the group with multitypic antibody responses: Non-patient 1 group was 6.52 ± 0.86 for DENV-1; 6.52 ± 0.347 for DENV-2; 5.12 ± 0.66 for DENV-3; 2.32 ± 0 for DENV-4. In contrast the means of N.A titers of serum samples in the patient group were 4.54 ± 0.69 for DENV-1; 5.1 ± 0.28 for DENV-2; 4.32 ± 0.47 for DENV-3; 3.32 ± 0.47 for DENV-4 (Fig. 2c). The mean of N.A titer for DENV-2 in pre-dengue season group (Non patient 1) was significantly higher than that of the patient group (p = 0.01, Fig. 2c).

**Fig. 3:**
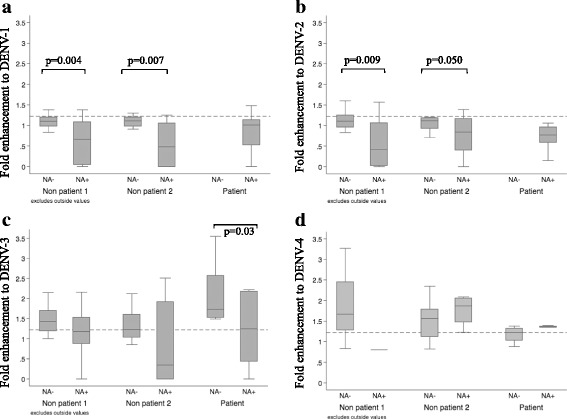
Fold-enhancement activity in 34 pair serum samples obtained from 34 individuals who were DENV IgG positive before the dengue season and demonstrated elevated DENV IgG after the season versus patient group. Fold enhancement value was determined by the ratio of (mean plaque count at 1:20 serum dilution)/(mean plaque count in the absence of human serum samples) by using FcγR expressing BHK cells. Dash line represents for cut-off plus 2 times SD value of infection-enhancement. Positive infection-enhancing activity was defined as fold enhancement value greater than cut-off value plus 2 times SD (above dash line). *P*-value was determined by Mann -Whitney test. *P*-value ≤0.05 = significant. NA-: Neutralizing antibody titer <10; NA+: Neutralizing antibody titer ≥10. Non patient 1 = pre-dengue season group; Non patient 2 = post-dengue season group; Patient = confirmed DENV infected patient group. The results were represented as Median ± interquartile range (Median ± IQR)

DENV enhancement activity was also determined by using FcγR-expressing BHK cells (Fig. 3). In the pre-dengue season group (Non patient 1 group), fold-enhancement to DENV-1 in samples with neutralizing activity against DENV-1 (N.A titers ≥10) was 0.66 ± 1.03 (Median ± IQR). In contrast, the DENV-1 fold-enhancement activity of those samples with no neutralizing antibody to DENV-1 (N.A titers <10) was 1.1 ± 0.22 (*p* = 0.004). Similarly, fold-enhancement to DENV-2 in samples with DENV-2 N.A titers ≥10 was 0.42 ± 1.04, and with DENV-2 N.A titers <10 was 1.1 ± 0.29 (*p* = 0.009). Fold enhancement to DENV-3 of serum samples with DENV-3 N.A titers ≥10 was 1.18 ± 0.65, while with DENV-3 N.A titers <10 was 1.43 ± 0.51, and fold enhancement to DENV-4 of serum samples with DENV-4 N.A titers ≥10 was 0.8 ± 0, while DENV-4 N.A titers <10 was 1.67 ± 1.17. Among the patient group, all serum samples had N.A (N.A titers ≥10) to DENV-1 and DENV-2; fold enhancement to DENV-3 of serum samples with DENV-3 N.A ≥ 10 was 1.24 ± 1.32 while DENV-3 N.A < 10 was 1.73 ± 1.03 (*p* = 0.03); fold enhancement to DENV-4 of serum samples with DENV-4 N.A ≥ 10 was 1.34 ± 0.03, while DENV-4 N.A < 10 was 1.22 ± 0.13. The results indicated that among healthy volunteers (Non patient 1 and Non patient 2 groups), serum samples with high levels of neutralizing antibody against DENV-1 or DENV-2 demonstrated lower levels of infection-enhancing activity (Fig. 3a, b).

### DENV-neutralizing antibody profiles and DENV infection-enhancement activity in possible DENV infection in pre-dengue season residents versus the acute secondary DENV infection patient group

**Fig. 4:**
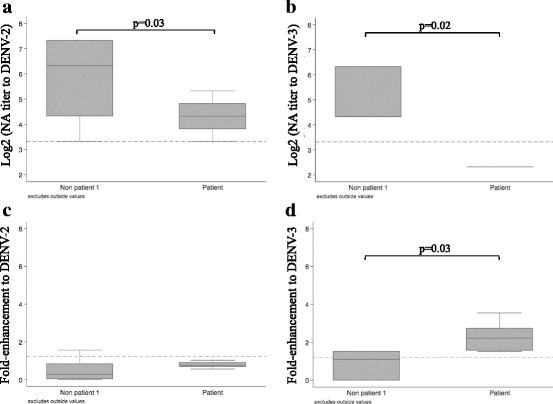
Comparison between possible past DENV infection in pre-exposure group vs. patient group for neutralizing antibody titer and fold enhancement. **a** & **c**: Non patient 1 = serum samples from pre-exposure group with possible infecting serotype of DENV-2 (based on the highest N.A titer reacts to DENV-2); Patient = serum samples from patient group with infecting serotype of DENV-2 (virology confirmation). **b** & **d**: Non patient 1 = serum samples from pre-exposure group with possible infecting serotype of DENV-3 (based on the highest N.A titer reacts to DENV-3); Patient = serum samples from patient group with infecting serotype of DENV3 (virology confirm). Fold enhancement value was determined by the ratio of (mean plaque count at 1:20 serum dilution)/(mean plaque count in the absence of human serum samples) by using FcγR-expressing BHK cells. Dash line represents for cut-off plus 2 times SD value of infection-enhancement. Positive infection-enhancing activity was defined as fold enhancement value greater than cut-off value plus 2 times SD (above dash line). *P*-value was determined by Mann-Whitney test. *P*-value ≤0.05 = significant. The results were represented as Median ± interquartile range (Median ± IQR)

In the group of individuals with elevated levels of DENV IgG (*N* = 34), before exposure to the dengue season, possible past DENV infection to specific serotype was determined based on the PRNT_50_ results (Additional file 2: Table S2). N.A titer to DENV-2 in serum samples of pre-dengue season group (Non patient 1) with possible prior exposure to DENV-2 was 6.32 ± 3 (median ± IQR), while N.A titer to DENV-2 in serum samples of the patient group (labeled as Patient) with infecting serotype DENV-2 was 4.32 ± 1 (*p* = 0.03) (Fig. 4a). N.A titer to DENV-3 in serum samples of the pre-dengue season group with prior exposure to DENV-3 was 4.32 ± 2, while N.A titer to DENV-3 in serum samples of the patient group with infecting serotype DENV-3 was 2.32 ± 0 (*p* = 0.02) (Fig. 4b). Serum samples of the pre-dengue season group with prior exposure to DENV-2 demonstrated fold-enhancement of 0.29 ± 0.78 (median ± IQR). In contrast, the patient group with DENV-2 infecting serotype demonstrated fold-enhancement of 0.77 ± 0.2 (*p* = 0.057) (Fig. 4c). Similarly, serum samples of the pre-dengue season group with prior DENV-3 exposure demonstrated fold-enhancement of 1.1 ± 1.5 to DENV-3. In contrast, the patient group with DENV-3 infecting serotype demonstrated DENV-3 fold-enhancement of 2.2 ± 1.16 (p = 0.03) (Fig. 4d). Concurring with the results of previous studies [6], while neutralizing antibodies were detected to infecting serotypes in 12 (12/20, 60%) samples using BHK cells (PRNT_50_ = 10 – 40), only 2 samples (2/20, 10%, coded as 133 and 355) demonstrated low levels of neutralizing activity (PRNT_50_ = 10) using FcγR-expressing cells (Additional file 2: Table S2, section B-Acute secondary DENV infection patients, the patients with underlined N.A).

Neutralizing and infection-enhancing activities from 18 new asymptomatic DENV infection cases (post-dengue season) versus confirmed DENV infection patient group are summarized in Additional file 2: Table S4, Additional file 1: Figures S2 and S3. At the end of the dengue season, at the time of getting the second serum samples for post-dengue season evaluation, all residents in this study were completely asymptomatic. Of 18 DENV new asymptomatic DENV infection cases (labeled as Non patient group in Additional file 1: Figures S2, S3), neutralizing antibodies for DENV-1 and DENV-2 were detected in BHK cells although the N.A titers were not as high as those of the patient group (Additional file 1: Figure S2A, Additional file 2: Table S4). Fold enhancement of these individuals with and without neutralizing antibody responses were also examined to determine the enhancing activity to four DENV serotypes (Additional file 1: Figure S3). Although the differences were not significant, some participants who had negative immune response (N.A (−) or PRNT_50_ < 10) demonstrated a tendency to have a higher level of fold enhancement than those samples that demonstrated neutralizing activity (N.A (+) or PRNT_50_ ≥ 10) to some specific DENV serotypes (Additional file 1: Figure S3, section A, C for Non patient group). Neutralizing antibody responses from participants having DENV IgM before and after the dengue season in this study were presented in Additional file 3: Table S5.

## Discussion

DENV is a major public health issue in Viet Nam and many parts of the world. DENV infections are reported cases, thus passive surveillance through case notification does not faithfully reflect the true dengue situation in a population because medical care is not sought for either mild-moderate or asymptomatic cases. Sero-prevalence, therefore could shed light on true dengue infections in the population. In this study, 34% (34/100) of seropositive individuals were found to have possible DENV exposure before the dengue season, although they did not have any symptoms/signs suggesting dengue fever within the three months before recruitment nor during the study period. After the dengue season, the IgG prevalence was elevated to 52% (52/100). These results indicated the high probability of individuals unaware of themselves getting DENV infection in this dengue endemic area. Furthermore, these results point out the importance of having a comprehensive assessment for asymptomatic cases in a population as they account for three-quarters of the dengue infections and are a major part of dengue burden [2, 26–28].

The prevalence of dengue IgG antibodies was the highest among residents aged 20-30 regardless of pre or post-exposure to the dengue season. Our finding was in agreement with Ghazi A [29] and Le Van Tuan [30] that this age group was the target population for dengue infection in adults. The dengue season impacted Ha Noi residents as post-dengue IgG titers were significantly higher than pre-dengue IgG titers in the whole study population (*p* = 0.01) (Table 2) as well as in the group of 34 individuals with elevated DENV IgG (*p* = 0.015, data not shown), although none of them was symptomatic.

In this study, our data is unique as it compares the levels of neutralizing antibody in the presence and the absence of FcγR on BHK cells. Neutralizing antibodies against four DENV serotypes were detected on BHK cells whether the residents were exposed or not to the dengue season during the study period (Additional file 2: Tables S1 & S4, Additional file 1: Figures S1 & S2). The percentages of the population having neutralizing antibodies reacting to DENV-1 and DENV-2 were substantial higher than other serotypes, particular for post-dengue season (Fig. 1a). The results indicate that these DENV serotypes were the predominant serotypes responsible for dengue season in Ha Noi in 2014 and 2015. These observations were in agreement with the sero-epidemiological data indicating that while all four DENV serotypes have been found co-circulating in Viet Nam, the dominant serotypes in the dengue season in 2014 and 2015 in Ha Noi were mainly driven by DENV-1 and DENV-2 [31]. In spite of having no symptoms, the study participants may represent the population in which some had protective immunity prior to the dengue season. Regarding the patient group, none of them was infected with DENV-1 (Additional file 2: Table S2, section B-Acute secondary DENV infection patients). Four DENV serotypes co-circulates in Viet Nam during the dengue season. Epidemiological data indicated that DENV-1 and -2 serotypes were the predominant circulating serotypes in 2014-2015. It is likely that while most of the dengue cases in Ha Noi were caused by DENV-1 and DENV-2, the probability of exposure to DENV-3 or DENV-4 was possible but lower as compared to that of DENV-1 and DENV-2. In the patient group, samples from patients who demonstrated symptoms ≤3 days after the onset of disease, positive for virus isolation and RT-PCR, as well as matching demographic characteristics were chosen. As such, only 20 DENV infection cases qualified to be in the patient group during the study period, leading to the difference in the DENV serotype proportion.

PRNT is a measurement of an antibody’s ability to inhibit infection either by blocking virus entry or preventing virus uncoating [32, 33]. The present study found that PRNT performed on BHK cells (also known as FcγR-negative BHK or non-FcγR BHK cells) showed higher neutralizing antibodies (N.A) titers than those on FcγR-expressing BHK cells (Additional file 1: Figures S1 & S2) and in some samples, N.A titers were only detected on FcγR-expressing BHK cells (Additional file 2: Tables S1 & S4). Generally, Vero cells and BHK cells are used as assay cells for conventional PRNT. However, these cells do not express the FcγR. Thus, in the absence of FcγR, conventional PRNT exclusively detects neutralizing activity of an antibody, but not the infection-enhancement activity. Because the primary targets of DENV infection are FcγR-bearing monocytes and macrophages [34, 35], FcγR-expressing BHK cells could better reflect the biological properties of an antibody [6]. Although monocyte-lineage cells have been useful in determining infection-enhancement activity, the FcγR-expressing cells offer a platform in determining and comparing neutralizing activity in the presence or absence of the receptor. In DENV infection, it is generally accepted that individuals with heterotypic secondary DENV infection have higher risk of developing severe dengue compared to primary infection. During secondary infection, weakly neutralizing antibodies from the first infection bind to the second serotype and enhance infection of FcγR bearing myeloid cells such as monocytes and macrophages, in a phenomenon known as antibody-dependent enhancement (ADE) [8, 36–38]. It is known that the levels of neutralizing antibody titers are inversely associated with the infection-enhancement activities. Given that neutralizing antibodies play a central role in disease protection and in defining disease outcome, determination of the balance of the neutralizing and infection-enhancement activity in non-patients would be key to understanding the biological relevance of sub-neutralizing ADE antibodies.

In this study, the non-patient groups demonstrated higher levels of N.A titers in both BHK and FcγR-expressing BHK cells compared to the patient group (Additional file 1: Figures S1A & S1B). In addition, in the group with heterotypic neutralizing antibody responses, N.A titers to DENV-2 and DENV-3 in pre-dengue season (Non patient 1 group) were significantly higher than those of the patient group (Fig. 4a, b and Additional file 2: Table S2). The degree of fold enhancement to DENV-2 and DENV-3 in the Non-patient 1 group was also lower than those of the patient group (Fig. 4c, d and Additional file 2: Table S3). The same comparison was not performed for DENV-1 and DENV-4 as DENV-1 infection was not confirmed in the patient group. Additionally, with possible DENV-4 exposure among the group of 34 residents who had IgG seropositive before the season (Additional file 2: Table S2, section A-Non patient group before dengue season) for Non patient 1 group was not detected. Further studies using a larger sample size for both the non-patient groups and patient group would better define the immune mechanisms of N.A activity and fold-enhancement levels among individuals with heterotypic neutralizing antibody responses. The results suggested that in non-patients with multitypic DENV responses: (1) serum samples with high neutralizing activity exhibited no or low levels of infection enhancement activity, and (2) neutralizing activity in the presence of FcγR was higher compared to the patient group (Fig. 4, Additional file 2: Table S2). Taken together, the results of this study support the hypothesis that DENV serotype cross-reactive immunity induced by infection plays two competing roles: (1) at neutralizing level the absence of infection-enhancement activity facilitates disease protection leading to absence of symptoms or milder disease outcomes, and (2) infection-enhancement activity abrogates neutralizing activity, potentially leading to symptomatic infection and severe clinical presentations.

## Conclusions

In summary, serum samples from the healthy volunteers with no clinical DENV symptoms demonstrated high levels of neutralizing antibodies and low or absence of infection-enhancement antibodies. Our study highlighted that while ADE activity hampers the neutralizing activity of antibodies, high levels of neutralizing DENV antibodies set a critical threshold that facilitates the prevention of disease progression. Studies on DENV asymptomatic infections therefore are essential to provide insight on disease epidemiology and vaccine development; particularly on neutralizing antibody responses to DENV infection that lead to disease protection.

## Additional files

Additional file 1: Figure S1. Neutralizing antibody (N.A) profile in 34 pair serum samples obtained from 34 individuals who were DENV IgG positive pre-dengue season and demonstrated elevated DENV IgG after the season versus patient group. Neutralizing activity was determined by PRNT_50_ on BHK cells (**A**) and on FcγR-expressing BHK cells (**B**); Positive PRNT samples were defined as having a N.A ≥ 10 or Log_2_(PRNT_50_) ≥3.32 to any of the virus test (above dotted line). Non patient 1=pre-dengue season group; Non patient 2 = post-dengue season group; Patient = patient group. The results were represented as means of Log_2_ (N.A titers) ± SD. (PDF 314 kb)

Additional file 2: Figure S2. Neutralizing antibody (N.A) profile in serum samples obtained from 18 individuals who were DENV IgG negative pre-dengue season but seropositive post-dengue season (Non patient) versus patient group. Neutralizing activity was determined by PRNT_50_ on BHK (A) and on FcγR-expressing BHK (B). Positive PRNT samples were defined as having a N.A ≥ 10 or Log_2_ (PRNT_50_) ≥3.32 to any of the virus test (above dotted line). The results were represented as means of Log_2_ (N.A titers) ± SD. (DOCX 55 kb)

Additional file 3: Figure S3. Fold-enhancement activity in serum samples obtained from 18 individuals who were DENV IgG negative pre-dengue season but seropositive post-dengue season (Non patient group) versus patient group. Fold- enhancement value was determined by the ratio of (mean plaque count at 1:20 serum dilution)/(mean plaque count in the absence of human serum samples) by using FcγR expressing BHK cells. Positive infection-enhancing activity was defined as fold-enhancement value greater than cut-off value plus 2 times SD (above dash line). *P*-value was determined by Mann -Whitney test. *P* value £0.05 = significant. Non patient = Non patient group; Patient = Patient group. NA(-): Neutralizing antibody titer <10; NA(+): Neutralizing antibody titer ≥ 10. The results were represented as Median ±IQR. (DOCX 21 kb)

## Abbreviations

ADE: Antibody dependent enhancement
BHK: Baby hamster kidney
C.I: Confidence interval
DENV: Dengue virus
DHF: Dengue hemorrhagic fever
ELISA: enzyme-linked immunosorbent assay
EMEM: Eagle’s minimum essential medium
FCS: Fetal calf serum
FcγR-expressing BHK cell line: Fcγ Receptor-expressing baby hamster kidney cell line
HRPO: Horseradish peroxidase
ICF: Infected culture fluid
IgG: Immunoglobulin G
IgM: Immunoglobulin M
IQR: Interquartile range
N.A titer: Neutralizing antibody titer
OPD: O-Phenylenediamin dihydrochloride
OR: Odd Ratio
PBS-T: Phosphate buffered saline containing 0.05% tween 20
PFU: Plaque forming unit
PRNT: Plaque reduction neutralization test
PRNT_50_: Fifty percent plaque reduction neutralization test
RT: Room temperature
SD: Standard deviation
