# Supplementary figures and images for: Dengue virus infection-enhancement activity in neutralizing antibodies of healthy adults before dengue season as determined by using FcγR-expressing cells
Source: BMC Infect Dis. 2018 Jan 10;18:31. doi: 10.1186/s12879-017-2894-7 (PMC5763606; doi:10.1186/s12879-017-2894-7)

Figure S1

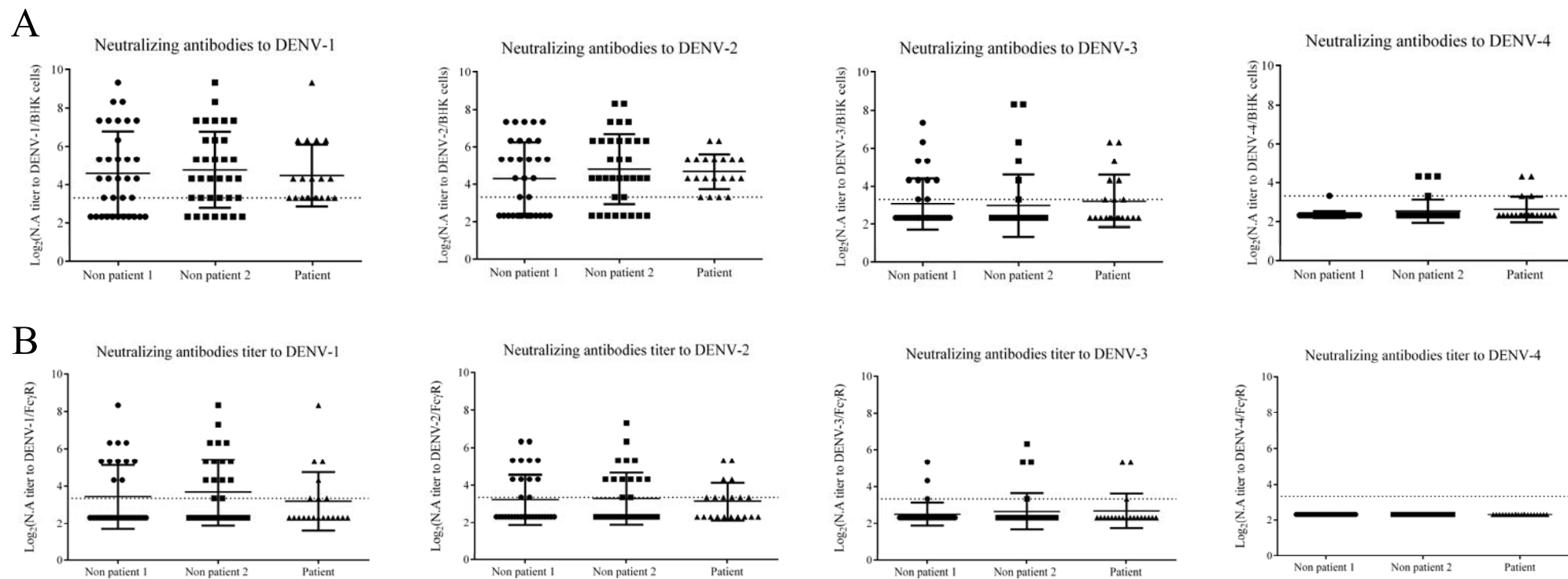

Figure S2

A

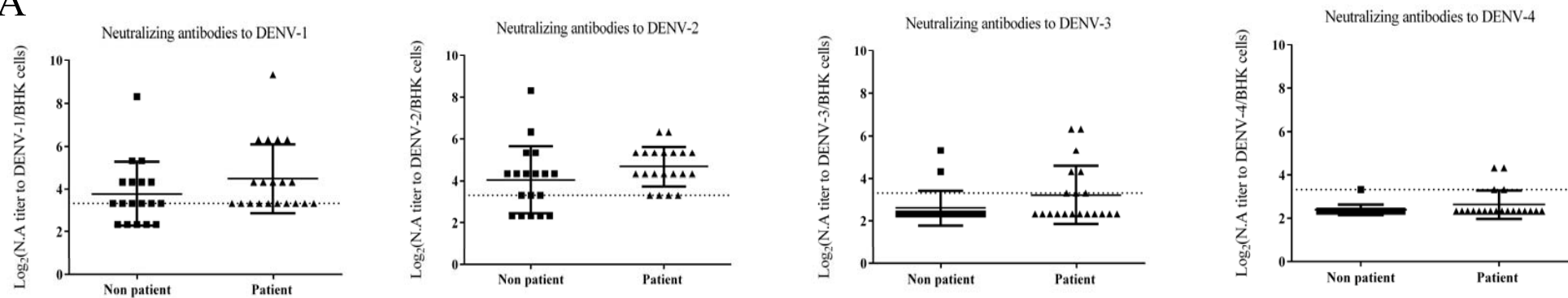

B

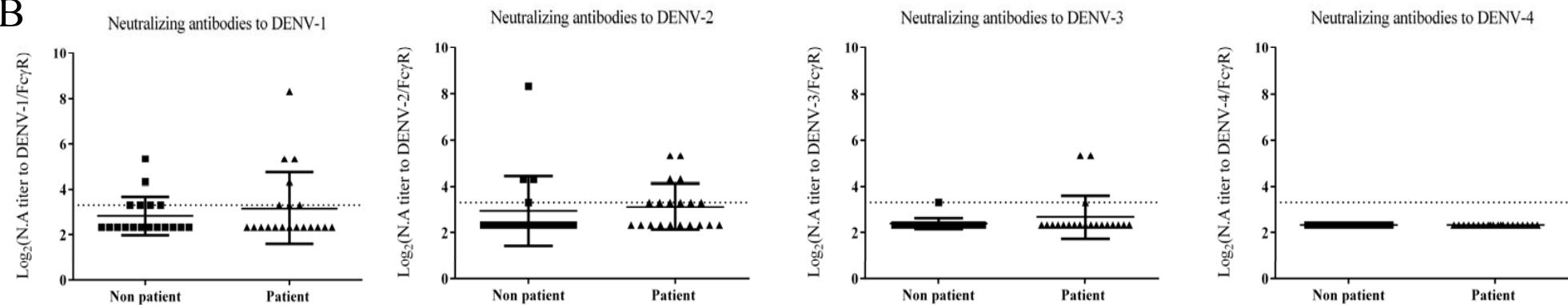

Figure S3

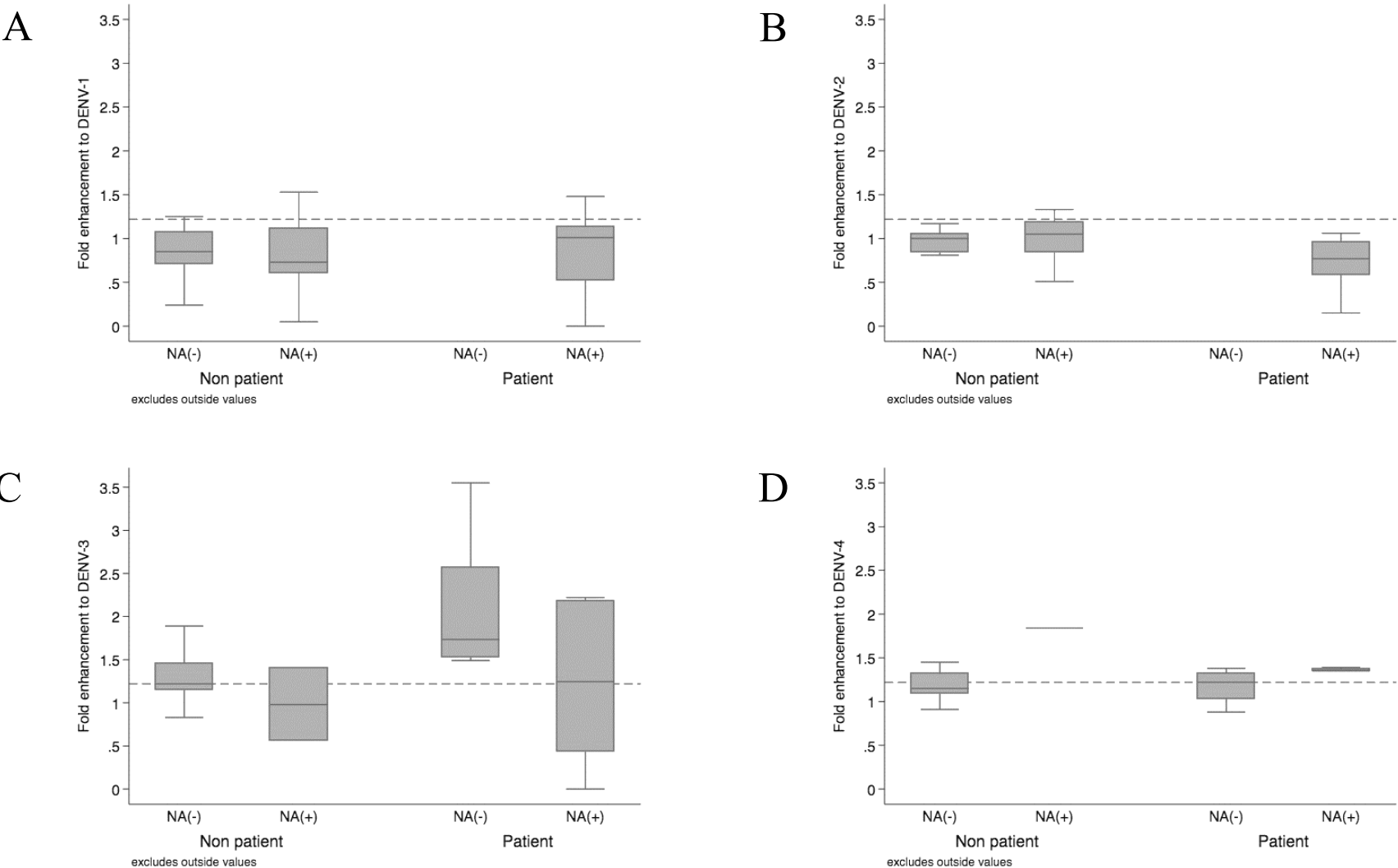

Supplement: Supplementary file 1 — Neutralizing antibody (N.A) profile in 34 pair serum samples obtained from 34 individuals who were DENV IgG positive pre-dengue season and demonstrated elevated DENV IgG after the season versus patient group. Neutralizing activity was determined by PRNT50 on BHK cells (A) and on FcγR-expressing BHK cells (B); Positive PRNT samples were defined as having a N.A ≥ 10 or Log2(PRNT50) ≥3.32 to any of the virus test (above dotted line). Non patient 1=pre-dengue season group; Non patient 2 = post-dengue season group; Patient = patient group. The results were represented as means of Log2 (N.A titers) ± SD. (PDF 314 kb) [file 12879_2017_2894_MOESM1_ESM.pdf]
