# Supplementary material for: Dengue virus infection-enhancement activity in neutralizing antibodies of healthy adults before dengue season as determined by using FcγR-expressing cells
Source: BMC Infect Dis. 2018 Jan 10;18:31. doi: 10.1186/s12879-017-2894-7 (PMC5763606; doi:10.1186/s12879-017-2894-7)
Supplement: Supplementary file 2 — Neutralizing antibody (N.A) profile in serum samples obtained from 18 individuals who were DENV IgG negative pre-dengue season but seropositive post-dengue season (Non patient) versus patient group. Neutralizing activity was determined by PRNT50 on BHK (A) and on FcγR-expressing BHK (B). Positive PRNT samples were defined as having a N.A ≥ 10 or Log2 (PRNT50) ≥3.32 to any of the virus test (above dotted line). The results were represented as means of Log2 (N.A titers) ± SD. (DOCX 55 kb) [file 12879_2017_2894_MOESM2_ESM.docx]

Additional file 2

Table S1: Neutralizing antibody titer (PRNT_50_) to DENV in 34 individuals (non-patient group) that demonstrated an increase in anti-DENV IgG antibodies levels.

| **Code** | **Before dengue season** | | | | | | | | | | | | | | **After dengue season** | | | | | | | | | | | | | | | |
| --- | --- | --- | --- | --- | --- | --- | --- | --- | --- | --- | --- | --- | --- | --- | --- | --- | --- | --- | --- | --- | --- | --- | --- | --- | --- | --- | --- | --- | --- | --- |
|  | **BHK cell line (PRNT_50_^a^)** | | | | | | | **FcγR-expressing BHK cell line (PRNT_50_)** | | | | | | | **BHK cell line (PRNT_50_)** | | | | | | | | **FcγR-expressing BHK cell line (PRNT_50_)** | | | | | | | |
|  | **DENV-1** | | **DENV-2** | | **DENV-3** | **DENV-4** | **DENV-1** | | | **DENV-2** | | **DENV-3** | | **DENV-4** | | **DENV-1** | | **DENV-2** | | **DENV-3** | | **DENV-4** | | **DENV-1** | | **DENV-2** | | **DENV-3** | | **DENV-4** |
| HN15.H.001^+^ | <10 | <10 | | 80 | | <10 | | <10 | <10 | | 40 | | <10 | | 20 | | 20 | | 320 | | <10 | | <10 | | <10 | | 80 | | <10 | |
| HN15.H.004 | 40 | 20 | | <10 | | <10 | | 20 | <10 | | <10 | | <10 | | 40 | | 20 | | <10 | | <10 | | 20 | | <10 | | <10 | | <10 | |
| HN15.H.005 | 160 | 10 | | <10 | | <10 | | 40 | <10 | | <10 | | <10 | | 160 | | 20 | | <10 | | <10 | | 40 | | <10 | | <10 | | <10 | |
| HN15.H.010 | 80 | 160 | | 160 | | <10 | | 40 | 80 | | 20 | | <10 | | 160 | | 320 | | 80 | | 20 | | 40 | | 160 | | 40 | | <10 | |
| HN15.H.011 | 640 | 40 | | 10 | | <10 | | 320 | 20 | | <10 | | <10 | | 640 | | 40 | | 10 | | <10 | | 320 | | 20 | | <10 | | <10 | |
| HN15.H.013^+^ | <10 | 20 | | <10 | | <10 | | <10 | <10 | | <10 | | <10 | | <10 | | 80 | | <10 | | <10 | | <10 | | 40 | | <10 | | <10 | |
| HN15.H.016 | <10 | <10 | | 20 | | <10 | | <10 | <10 | | <10 | | <10 | | 40 | | <10 | | <10 | | <10 | | 10 | | <10 | | <10 | | <10 | |
| HN15.H.018 | <10 | <10 | | 20 | | <10 | | <10 | <10 | | <10 | | <10 | | 10 | | 20 | | <10 | | 20 | | <10 | | <10 | | <10 | | <10 | |
| HN15.H.020 | <10 | <10 | | 20 | | <10 | | <10 | <10 | | <10 | | <10 | | 10 | | 20 | | <10 | | <10 | | <10 | | <10 | | <10 | | <10 | |
| HN15.H.021^+^ | <10 | 40 | | <10 | | <10 | | <10 | 10 | | <10 | | <10 | | <10 | | 80 | | <10 | | <10 | | <10 | | 40 | | <10 | | <10 | |
| HN15.H.022 | 20 | 80 | | 40 | | <10 | | <10 | 40 | | <10 | | <10 | | 40 | | 80 | | <10 | | <10 | | <10 | | 20 | | <10 | | <10 | |
| HN15.H.023 | <10 | <10 | | <10 | | <10 | | <10 | <10 | | <10 | | <10 | | <10 | | <10 | | <10 | | <10 | | <10 | | <10 | | <10 | | <10 | |
| HN15.H.024 | 10 | <10 | | 10 | | <10 | | <10 | <10 | | <10 | | <10 | | 20 | | <10 | | <10 | | <10 | | <10 | | <10 | | <10 | | <10 | |
| HN15.H.026 | 40 | 160 | | <10 | | <10 | | <10 | 20 | | <10 | | <10 | | 20 | | 160 | | <10 | | <10 | | <10 | | 20 | | <10 | | <10 | |
| HN15.H.027^+^ | 10 | <10 | | <10 | | <10 | | <10 | <10 | | <10 | | <10 | | <10 | | 80 | | 320 | | <10 | | <10 | | 10 | | 40 | | <10 | |
| HN15.H.031 | <10 | <10 | | <10 | | 10 | | <10 | <10 | | <10 | | <10 | | <10 | | <10 | | <10 | | 10 | | <10 | | <10 | | <10 | | <10 | |
| HN15.H.037 | 40 | 80 | | 20 | | <10 | | 20 | 40 | | <10 | | <10 | | 40 | | 40 | | <10 | | <10 | | 20 | | 20 | | <10 | | <10 | |
| HN15.H.039 | 320 | <10 | | <10 | | <10 | | 80 | <10 | | <10 | | <10 | | 160 | | <10 | | <10 | | <10 | | 80 | | <10 | | <10 | | <10 | |
| HN15.H.041 | 160 | 160 | | 40 | | <10 | | 80 | 80 | | 10 | | <10 | | 160 | | 160 | | 40 | | <10 | | 80 | | 80 | | 10 | | <10 | |
| HN15.H.042^+^ | <10 | 10 | | <10 | | <10 | | <10 | <10 | | <10 | | <10 | | 320 | | 320 | | 20 | | 20 | | 160 | | 40 | | <10 | | <10 | |
| HN15.H.047 | 10 | <10 | | <10 | | <10 | | <10 | <10 | | <10 | | <10 | | 10 | | <10 | | <10 | | <10 | | <10 | | <10 | | <10 | | <10 | |
| HN15.H.049 | 20 | 160 | | <10 | | <10 | | <10 | 40 | | <10 | | <10 | | <10 | | 80 | | <10 | | <10 | | <10 | | 20 | | <10 | | <10 | |
| HN15.H.052 | <10 | <10 | | <10 | | <10 | | <10 | <10 | | <10 | | <10 | | 10 | | 20 | | <10 | | <10 | | <10 | | <10 | | <10 | | <10 | |
| HN15.H.056 | 40 | <10 | | <10 | | <10 | | <10 | <10 | | <10 | | <10 | | 20 | | 10 | | <10 | | <10 | | <10 | | <10 | | <10 | | <10 | |
| HN15.H.068 | 20 | 80 | | <10 | | <10 | | <10 | 20 | | <10 | | <10 | | 20 | | 80 | | <10 | | <10 | | 10 | | <10 | | <10 | | <10 | |
| HN15.H.071 | 10 | 80 | | <10 | | <10 | | <10 | 10 | | <10 | | <10 | | <10 | | 80 | | <10 | | <10 | | <10 | | 10 | | <10 | | <10 | |
| HN15.H.080 | 160 | 40 | | <10 | | <10 | | 40 | <10 | | <10 | | <10 | | 80 | | 20 | | <10 | | <10 | | 40 | | <10 | | <10 | | <10 | |
| HN15.H.082 | 160 | 40 | | <10 | | <10 | | 40 | <10 | | <10 | | <10 | | 80 | | 20 | | <10 | | <10 | | 40 | | <10 | | <10 | | <10 | |
| HN15.H.084 | 160 | <10 | | <10 | | <10 | | 80 | <10 | | <10 | | <10 | | 160 | | <10 | | <10 | | <10 | | 80 | | <10 | | <10 | | <10 | |
| HN15.H.086 | 40 | 160 | | <10 | | <10 | | <10 | 40 | | <10 | | <10 | | 10 | | 160 | | <10 | | <10 | | <10 | | 20 | | <10 | | <10 | |
| HN15.H.089 | 20 | <10 | | <10 | | <10 | | <10 | <10 | | <10 | | <10 | | 40 | | <10 | | <10 | | <10 | | 20 | | <10 | | <10 | | <10 | |
| HN15.H.091 | 20 | 40 | | <10 | | <10 | | <10 | <10 | | <10 | | <10 | | 20 | | 20 | | <10 | | <10 | | <10 | | <10 | | <10 | | <10 | |
| HN15.H.097 | 320 | 40 | | <10 | | <10 | | 40 | 20 | | <10 | | <10 | | 80 | | 40 | | <10 | | <10 | | 20 | | <10 | | <10 | | <10 | |
| HN15.H.099 | <10 | 20 | | <10 | | <10 | | <10 | <10 | | <10 | | <10 | | 10 | | 10 | | <10 | | <10 | | <10 | | <10 | | <10 | | <10 | |

^a^ Neutralizing antibody titer (PRNT_50_) indicates end point at which the highest serum dilution inhibited ≥ 50% the plaques. Positive samples were defined as those that demonstrated neutralizing antibody titers of 10 or above to any of serotypes (PRNT_50_ ≥10). A titer of 10 or below (PRNT_50_<10) indicates neutralizing antibody that were below detection levels. Plus sign (+) indicates samples with 4-fold increase of neutralizing antibody titer after the dengue season by using BHK and FcγR-expressing BHK cells.

Table S2: Levels of neutralizing activity in serum samples obtained from healthy residents before exposure to dengue season and from secondary acute dengue infection patients against each of the 4 DENV serotypes.

| **CODE** | **BHK cell line (PRNT_50_^a^)** | | | | **FcγR-expressing BHK cell line (PRNT_50_^a^)** | | | |  |
| --- | --- | --- | --- | --- | --- | --- | --- | --- | --- |
|  | **DENV-1** | **DENV-2** | **DENV-3** | **DENV-4** | **DENV-1** | **DENV-2** | **DENV-3** | **DENV-4** |  |
| **A. Non-patient group before dengue season** |  |  |  |  |  |  |  |  | **Possible DENV exposure^*^** |
| HN15.H.005/1 | 160 | 10 | <10 | <10 | 40 | <10 | <10 | <10 | DENV-1 |
| HN15.H.011/1 | 640 | 40 | 10 | <10 | 320 | 20 | <10 | <10 | DENV-1 |
| HN15.H.039/1 | 320 | <10 | <10 | <10 | 80 | <10 | <10 | <10 | DENV-1 |
| HN15.H.056/1 | 40 | <10 | <10 | <10 | <10 | <10 | <10 | <10 | DENV-1 |
| HN15.H.080/1 | 160 | 40 | <10 | <10 | 40 | <10 | <10 | <10 | DENV-1 |
| HN15.H.082/1 | 160 | 40 | <10 | <10 | 40 | <10 | <10 | <10 | DENV-1 |
| HN15.H.084/1 | 160 | <10 | <10 | <10 | 80 | <10 | <10 | <10 | DENV-1 |
| HN15.H.089/1 | 20 | <10 | <10 | <10 | <10 | <10 | <10 | <10 | DENV-1 |
| HN15.H.097/1 | 320 | 40 | <10 | <10 | 40 | 20 | <10 | <10 | DENV-1 |
| HN15.H.013/1 | <10 | 20 | <10 | <10 | <10 | <10 | <10 | <10 | DENV-2 |
| HN15.H.021/1 | <10 | 40 | <10 | <10 | <10 | 10 | <10 | <10 | DENV-2 |
| HN15.H.022/1 | 20 | 80 | 40 | <10 | <10 | 40 | <10 | <10 | DENV-2 |
| HN15.H.026/1 | 40 | 160 | <10 | <10 | <10 | 20 | <10 | <10 | DENV-2 |
| HN15.H.042/1 | <10 | 10 | <10 | <10 | <10 | <10 | <10 | <10 | DENV-2 |
| HN15.H.049/1 | 20 | 160 | <10 | <10 | <10 | 40 | <10 | <10 | DENV-2 |
| HN15.H.068/1 | 20 | 80 | <10 | <10 | <10 | 20 | <10 | <10 | DENV-2 |
| HN15.H.071/1 | 10 | 80 | <10 | <10 | <10 | 10 | <10 | <10 | DENV-2 |
| HN15.H.086/1 | 40 | 160 | <10 | <10 | <10 | 40 | <10 | <10 | DENV-2 |
| HN15.H.099/1 | <10 | 20 | <10 | <10 | <10 | <10 | <10 | <10 | DENV-2 |
| HN15.H.001/1 | <10 | <10 | 80 | <10 | <10 | <10 | 40 | <10 | DENV-3 |
| HN15.H.016/1 | <10 | <10 | 20 | <10 | <10 | <10 | <10 | <10 | DENV-3 |
| HN15.H.018/1 | <10 | <10 | 20 | <10 | <10 | <10 | <10 | <10 | DENV-3 |
|  |  |  |  |  |  |  |  |  |  |
| **B. Acute secondary DENV infection patients** |  |  |  |  |  |  |  |  | **DENV infection^**^** |
| 22 | 80 | 40 | <10 | <10 | 20 | <10 | <10 | <10 | DENV-2 |
| 133 | 10 | 20 | 80 | <10 | <10 | 10 | 40 | <10 | DENV-2 |
| 256 | 10 | 10 | <10 | <10 | <10 | <10 | <10 | <10 | DENV-2 |
| 319 | 20 | 10 | <10 | <10 | <10 | <10 | <10 | <10 | DENV-2 |
| 355 | 10 | 40 | <10 | <10 | <10 | 10 | <10 | <10 | DENV-2 |
| 356 | 80 | 20 | <10 | <10 | 40 | <10 | <10 | <10 | DENV-2 |
| 357 | 10 | 20 | 20 | <10 | <10 | <10 | <10 | <10 | DENV-2 |
| 401 | 10 | 20 | <10 | <10 | 10 | <10 | <10 | <10 | DENV-2 |
| 235 | 20 | 10 | <10 | <10 | <10 | 10 | <10 | <10 | DENV-3 |
| 309 | 10 | 40 | <10 | <10 | <10 | 10 | <10 | <10 | DENV-3 |
| 348 | 10 | 10 | <10 | <10 | <10 | <10 | <10 | <10 | DENV-3 |
| 390 | 20 | 20 | <10 | <10 | <10 | <10 | <10 | <10 | DENV-3 |
| 559 | 20 | 20 | 10 | 10 | <10 | <10 | <10 | <10 | DENV-3 |
| 129 | 80 | 80 | 80 | <10 | 40 | 40 | 40 | <10 | DENV-4 |
| 334 | 80 | 40 | <10 | <10 | <10 | 20 | <10 | <10 | DENV-4 |
| 337 | 20 | 40 | 10 | <10 | <10 | 20 | <10 | <10 | DENV-4 |
| 386 | 640 | 20 | 20 | <10 | 320 | <10 | <10 | <10 | DENV-4 |
| 573 | 10 | 80 | 40 | 20 | 10 | 40 | 10 | <10 | DENV-4 |
| 578 | 10 | 40 | <10 | 20 | 10 | 10 | <10 | <10 | DENV-4 |
| 402 | 10 | 40 | 10 | 10 | <10 | 10 | <10 | <10 | DENV-3 & DENV-4 |

Asterisk (*) indicate possible DENV serotype that the healthy residents group were exposed to before dengue season. This was determined based on the highest neutralizing antibody titer of the specific DENV serotype using serum samples from the first sample collection, indicating possible DENV serotype exposure prior to the start of the study. A total of 22 samples with equal or more than 4-fold N.A. to a single serotype was listed in the table. Only samples with Double asterisks (**) indicates the infecting DENV serotype of the patient group as determined by using virus isolation and real-time PCR. PRNT_50_ indicates plaque reduction neutralizing antibody titer end point at which the highest serum dilution inhibited ≥ 50% of the plaques.

Table S3: DENV infection-enhancement activity in serum samples obtained from healthy residents and from patients with acute secondary DENV infection.

| **CODE** | **Fold-enhancement^a^** | | | |  |
| --- | --- | --- | --- | --- | --- |
| **A. Non-patient group**  **(before dengue season)** | **DENV-1** | **DENV-2** | **DENV-3** | **DENV-4** | **Possible past exposure^*^** |
| HN15.H.005/1 | -^b^ | 0.99 | 1.50 | 1.00 | DENV-1 |
| HN15.H.011/1 | - | 0.40 | 1.26 | 0.95 | DENV-1 |
| HN15.H.039/1 | 0.05 | 1.12 | 1.25 | 2.57 | DENV-1 |
| HN15.H.056/1 | 1.19 | 1.60 | 1.75 | 1.83 | DENV-1 |
| HN15.H.080/1 | 0.05 | 0.97 | 1.81 | 1.71 | DENV-1 |
| HN15.H.082/1 | - | 1.13 | 1.83 | 2.45 | DENV-1 |
| HN15.H.084/1 | - | 1.37 | 1.46 | 1.85 | DENV-1 |
| HN15.H.089/1 | 0.85 | 0.96 | 1.24 | 1.29 | DENV-1 |
| HN15.H.097/1 | 0.15 | 0.43 | 1.18 | 6.12 | DENV-1 |
| HN15.H.013/1^+^ | 1.10 | 0.83 | 2.15 | 1.64 | DENV-2 |
| HN15.H.021/1^+^ | 0.98 | 0.34 | 2.09 | 1.28 | DENV-2 |
| HN15.H.022/1 | 1.08 | 0.05 | 1.97 | 2.22 | DENV-2 |
| HN15.H.026/1 | 1.20 | - | 1.52 | 1.67 | DENV-2 |
| HN15.H.042/1^+^ | 1.00 | 1.57 | 1.11 | 0.90 | DENV-2 |
| HN15.H.049/1 | 0.93 | 0.10 | 2.50 | 4.70 | DENV-2 |
| HN15.H.068/1 | 0.83 | 0.64 | 1.44 | 1.83 | DENV-2 |
| HN15.H.071/1 | 1.15 | 0.23 | 1.16 | 4.91 | DENV-2 |
| HN15.H.086/1 | 1.00 | 0.00 | 1.33 | 1.64 | DENV-2 |
| HN15.H.099/1 | 0.93 | 1.54 | 1.36 | 4.22 | DENV-2 |
| HN15.H.001/1^+^ | 1.38 | 1.14 | - | 2.02 | DENV-3 |
| HN15.H.016/1 | 9.90 | 0.83 | 1.10 | 1.07 | DENV-3 |
| HN15.H.018/1 | 1.20 | 0.89 | 1.50 | 0.83 | DENV-3 |
|  |  |  |  |  |  |
| **B. Acute secondary**  **dengue patients** |  |  |  |  | **DENV infection^**^** |
| 22 | 0.48 | 0.65 | 1.49 | 0.88 | DENV-2 |
| 133 | 1.33 | 0.77 | - | 0.94 | DENV-2 |
| 256 | 1.48 | 0.92 | 1.96 | 1.28 | DENV-2 |
| 319 | 0.61 | 0.87 | 2.41 | 1.21 | DENV-2 |
| 355 | 1.13 | 0.57 | 1.59 | 1.23 | DENV-2 |
| 356 | - | 0.74 | 1.88 | 1.32 | DENV-2 |
| 357 | 1.14 | 1.03 | 1.00 | 0.44 | DENV-2 |
| 401 | 1.00 | 0.77 | 1.52 | 1.36 | DENV-2 |
| 235 | 1.11 | 1.06 | 1.51 | 1.13 | DENV-3 |
| 309 | 1.26 | 0.61 | 3.55 | 1.21 | DENV-3 |
| 348 | 1.02 | 1.00 | 2.73 | 1.23 | DENV-3 |
| 390 | 1.20 | 0.88 | 1.57 | 1.38 | DENV-3 |
| 559 | 1.13 | 1.00 | 2.22 | 1.35 | DENV-3 |
| 129 | - | 0.19 | - | 0.89 | DENV-4 |
| 334 | 0.89 | 0.29 | 3.11 | 1.21 | DENV-4 |
| 337 | 0.05 | 0.40 | 2.15 | 1.33 | DENV-4 |
| 386 | - | 0.85 | 1.49 | 1.37 | DENV-4 |
| 573 | 0.57 | 0.15 | 0.87 | 1.35 | DENV-4 |
| 578 | 0.72 | 0.71 | 1.54 | 1.39 | DENV-4 |
| 402 | 1.13 | 1.00 | 2.22 | 1.35 | DENV-3 &  DENV-4 |

^a^Fold enhancement value was determined by the ratio of (mean plaque count at 1:20 serum dilution)/(mean plaque count in the absence of human serum samples) by using FcγR-expressing BHK cells. Positive infection-enhancing activity (underline) was defined as fold infection-enhancement value of greater than cut-off value plus 2 times SD. ^b^ Minus sign (-) denotes no plaque detected at 1:20 serum dilution, indicating high levels of neutralizing antibodies. Cut-off value was determined in the absence of serum sample. Asterisk (*) indicate possible DENV serotype that the healthy residents group were exposed to before dengue season. This was determined based on the highest neutralizing antibody titer of the specific DENV serotype using serum samples from the first sample collection. A total of 22 samples with equal or more than 4-fold N.A. to a single serotype was listed in the table. Double asterisks (**) indicates the infecting DENV serotype of the patient group as determined by using virus isolation and real-time PCR. PRNT_50_ indicates plaque reduction neutralizing antibody titer end point at which the highest serum dilution inhibited ≥ 50% of the plaques.

Table S4: Dengue neutralizing antibody titers (PRNT_50_) in 18 individuals which demonstrated seroconversion after dengue season.

| **CODE** | **After dengue season** | | | | | | | |
| --- | --- | --- | --- | --- | --- | --- | --- | --- |
|  | **BHK cells (PRNT_50_^a^)** | | | | **FcγR-expressing BHK cells (PRNT_50_)** | | | |
|  | **DENV-1** | **DENV-2** | **DENV-3** | **DENV-4** | **DENV-1** | **DENV-2** | **DENV-3** | **DENV-4** |
| HN15.H.002 | <10 | 20 | 40 | <10 | <10 | <10 | 10 | <10 |
| HN15.H.003 | 20 | <10 | <10 | <10 | 10 | <10 | <10 | <10 |
| HN15.H.015 | 10 | 10 | <10 | <10 | <10 | <10 | <10 | <10 |
| HN15.H.019 | 20 | 320 | <10 | 10 | <10 | 80 | <10 | <10 |
| HN15.H.034 | 10 | 40 | <10 | <10 | <10 | 20 | <10 | <10 |
| HN15.H.059 | 40 | <10 | <10 | <10 | 40 | <10 | <10 | <10 |
| HN15.H.063 | 40 | 20 | <10 | <10 | 20 | <10 | <10 | <10 |
| HN15.H.064 | 10 | <10 | <10 | <10 | <10 | <10 | <10 | <10 |
| HN15.H.065 | <10 | 20 | <10 | <10 | <10 | <10 | <10 | <10 |
| HN15.H.066 | 320 | 20 | <10 | <10 | 40 | <10 | <10 | <10 |
| HN15.H.067 | 20 | <10 | <10 | <10 | 10 | <10 | <10 | <10 |
| HN15.H.069 | <10 | 40 | <10 | <10 | <10 | 10 | <10 | <10 |
| HN15.H.072 | 10 | 80 | <10 | <10 | <10 | 20 | <10 | <10 |
| HN15.H.077 | 10 | 20 | 20 | <10 | <10 | <10 | <10 | <10 |
| HN15.H.078 | <10 | 10 | <10 | <10 | <10 | <10 | <10 | <10 |
| HN15.H.079 | 10 | 20 | <10 | <10 | <10 | <10 | <10 | <10 |
| HN15.H.081 | 20 | <10 | <10 | <10 | 10 | <10 | <10 | <10 |
| HN15.H.090 | <10 | 10 | <10 | <10 | <10 | <10 | <10 | <10 |

^a^PRNT_50_ indicates the end point at which the highest serum dilution inhibited ≥ 50% the plaques. Positive PRNT samples were defined as having neutralizing antibody titer of 10 or above to any of the dengue serotypes tested (PRNT_50_ ≥10). PRNT_50_<10 indicates that there neutralizing antibody against DENV were below detection levels and PRNT_50_ ≥10 indicates presence of neutralizing antibody to DENV.
