# Supplementary material for: Dengue virus infection-enhancement activity in neutralizing antibodies of healthy adults before dengue season as determined by using FcγR-expressing cells
Source: BMC Infect Dis. 2018 Jan 10;18:31. doi: 10.1186/s12879-017-2894-7 (PMC5763606; doi:10.1186/s12879-017-2894-7)
Supplement: Supplementary file 3 — Fold-enhancement activity in serum samples obtained from 18 individuals who were DENV IgG negative pre-dengue season but seropositive post-dengue season (Non patient group) versus patient group. Fold- enhancement value was determined by the ratio of (mean plaque count at 1:20 serum dilution)/(mean plaque count in the absence of human serum samples) by using FcγR expressing BHK cells. Positive infection-enhancing activity was defined as fold-enhancement value greater than cut-off value plus 2 times SD (above dash line). P-value was determined by Mann -Whitney test. P value £0.05 = significant. Non patient = Non patient group; Patient = Patient group. NA(-): Neutralizing antibody titer <10; NA(+): Neutralizing antibody titer ≥ 10. The results were represented as Median ±IQR. (DOCX 21 kb) [file 12879_2017_2894_MOESM3_ESM.docx]

Additional file 3

Table S5a: Neutralization antibody titers from 6 sera had DENV IgM positive only before the dengue season in 2015

| No. | Code | DENV Elisa | | PRNT_50_ | | | | | | | |
| --- | --- | --- | --- | --- | --- | --- | --- | --- | --- | --- | --- |
|  |  |  |  | BHK cells | | | | FcγR-expressing BHK cells | | | |
|  |  | IgM_1_ | IgG_1_ | D1 | D2 | D3 | D4 | D1 | D2 | D3 | D4 |
| 1 | HN15.H.034 | (+) | (-) | <10 | 80 | <10 | <10 | <10 | 40 | <10 | <10 |
| 2 | HN15.H.040 | (+) | (-) | <10 | <10 | <10 | <10 | <10 | <10 | <10 | <10 |
| 3 | HN15.H.045 | (+) | (-) | <10 | <10 | <10 | <10 | <10 | <10 | <10 | <10 |
| 4 | HN15.H.064 | (+) | (-) | <10 | <10 | <10 | <10 | <10 | <10 | <10 | <10 |
| 5 | HN15.H.065 | (+) | (-) | 160 | 10 | <10 | <10 | 40 | <10 | <10 | <10 |
| 6 | HN15.H.079 | (+) | (-) | 20 | 40 | <10 | <10 | <10 | <10 | <10 | <10 |

IgM_1_: DENV IgM ELISA from serum samples before the dengue season.

IgG_1_: DENV IgG ELISA from serum samples before the dengue season.

Table S5b: Neutralization antibody titer from 6 sera had DENV IgM positive only after the dengue season in 2015

| No. | Code | DENV Elisa | | PRNT_50_ | | | | | | | |
| --- | --- | --- | --- | --- | --- | --- | --- | --- | --- | --- | --- |
|  |  |  |  | BHK cells | | | | FcγR-expressing BHK cells | | | |
|  |  | IgM_2_ | IgG_2_ | D1 | D2 | D3 | D4 | D1 | D2 | D3 | D4 |
| 1 | HN15.H.025 | (+) | (-) | <10 | 10 | 10 | <10 | <10 | <10 | <10 | <10 |
| 2 | HN15.H.033 | (+) | (-) | <10 | <10 | <10 | <10 | <10 | <10 | <10 | <10 |
| 3 | HN15.H.038 | (+) | (-) | <10 | <10 | <10 | <10 | <10 | <10 | <10 | <10 |
| 4 | HN15.H.040 | (+) | (-) | <10 | <10 | <10 | <10 | <10 | <10 | <10 | <10 |
| 5 | HN15.H.048 | (+) | (-) | <10 | 10 | <10 | <10 | <10 | <10 | <10 | <10 |
| 6 | HN15.H.058 | (+) | (-) | 160 | 10 | <10 | <10 | 10 | <10 | <10 | <10 |

IgM_2_: DENV IgM ELISA from serum samples after the dengue season.

IgG_2_: DENV IgG ELISA from serum samples after the dengue season.
